# The Impact of Prostate-Specific Antigen and Gleason Scores on Cardiovascular Death in Prostate Cancer Patients after Radiotherapy or Chemotherapy: A Population-Based Study

**DOI:** 10.31083/RCM24940

**Published:** 2025-02-19

**Authors:** Huijuan He, Liyu Guo, Peipei Wang, Yuting Yang, Zhenxing Lu, Xiaoping Peng, Tianwang Guan

**Affiliations:** ^1^The Second Clinical Medical College, Southern Medical University, 510280 Guangzhou, Guangdong, China; ^2^Department of Cardiology, The First Affiliated Hospital of Nanchang University, 330000 Nanchang, Jiangxi, China; ^3^Guangdong Engineering Research Center of Boron Neutron Therapy and Application in Malignant Tumors, Dongguan Key Laboratory of Precision Diagnosis and Treatment for Tumors, Dongguan Engineering Research Center for Innovative Boron Drugs and Novel Radioimmune Drugs, Cancer Center, The 10th Affiliated Hospital of Southern Medical University (Dongguan People’s Hospital), Southern Medical University, Guangdong 523059, China

**Keywords:** cardio-oncology, cardiovascular death, Gleason score, prostate cancer, prostate-specific antigen

## Abstract

**Background:**

Tumor characteristics are associated with the risk of cardiovascular death (CVD) in cancer patients. However, the influence of tumor characteristics on CVD risk among prostate cancer (PC) patients who have received radiotherapy (RT) or chemotherapy (CT) is often overlooked. This study explored the association between PC tumor characteristics and CVD risk in PC patients who had received RT or CT.

**Methods:**

Fine-gray competitive risk analysis was employed to identify CVD risk factors. Sensitivity analyses were conducted to adjust for confounding factors. The predicted prostate-specific antigen (PSA) and Gleason score values were visualized using a nomogram, which was subsequently validated through calibration curves and concordance indexes (C-indexes).

**Results:**

A total of 120,908 patients were enrolled in the study, with a mean follow-up time of 80 months. PSA values between 10 and 20 ng/mL (adjusted hazard ratio (HR): 1.28, 95% confidence interval (CI): 1.20–1.36, *p* < 0.001) and >20 ng/mL (adjusted HR: 1.27, 95% CI: 1.21–1.35, *p* < 0.001), and a Gleason score >7 (adjusted HR: 1.23, 95% CI: 1.07–1.41, *p* = 0.004) were identified as risk factors of CVD for PC patients after RT or CT. The C-index of the training cohort was 0.66 (95% CI: 0.66–0.67), and the C-index of the validation cohort was 0.67 (95% CI: 0.65–0.68). Consistency was observed between the actual observations and the nomogram. Risk stratification was also significant (*p* < 0.001).

**Conclusions:**

PSA values ≥10 ng/mL and Gleason scores >7 may be associated with an increased risk of CVD in PC patients after RT or CT. These patients may require more long-term follow-up and monitoring of CVD risk.

## 1. Introduction

Prostate cancer (PC) has become the most universal cancer among males in the United States. The number of new PC cases is projected to reach 299,010 in 2024, accounting for 29% of new cancer cases in the United States [1]. With the popularization of tumor screening and the advancement of treatment technology, more PC patients are diagnosed and treated at earlier stages, significantly improving patient survival rates [1]. Cardiovascular death (CVD) has increasingly become an important ingredient in the prognosis of cancer survivors. In previous research, PC patients were considered to have a higher incidence of cardiovascular disease, while cardiovascular events have been confirmed as the second leading cause of death in PC patients [2, 3]. Furthermore, the extensive use of anticancer treatments such as radiotherapy (RT) or chemotherapy (CT) will markedly elevate the risk of pre-existing CVD and diminish the overall survival of PC patients [2, 4, 5, 6, 7]. Therefore, identifying CVD risk factors and predicting the CVD risk for PC patients undergoing RT or CT is significant. Predicting risk factors is consequential in guiding clinical adjustments to treatment plans and implementing preventive measures promptly.

The risk of CVD in PC patients following RT or CT has mostly been studied for traditional risk factors and biological mechanisms shared by both CVD and cancer. These factors include obesity, high blood pressure, smoking, and neutrophil extracellular traps. Inflammation and oxidative stress are the mechanisms through which these factors lead to CVD and cancer [6, 8, 9, 10]. However, emerging research indicates a new view that the characteristics of the tumor itself are associated with CVD risk [11]. It remains unclear which tumor characteristics determine CVD risk and how they impact prognosis in PC patients.

To address this issue, we conducted a retrospective study to comprehensively analyze the influence of clinical and pathological features of tumors on the CVD risk of PC patients after RT or CT. We use nomograms to quantify and visualize results, identify CVD risk factors, and predict the CVD risk for PC patients after RT or CT from a novel perspective. It provides valuable insights for clinical monitoring of CVD risk, individualized, precise medicine for patients, and improving patient prognosis.

## 2. Materials and Methods

### 2.1 Data Source

The researched data were derived from the Surveillance, Epidemiology, and End Results (SEER) database. SEER is a nationally representative data system for the United States, covering approximately 30% of the population [6, 12, 13]. Information in SEER is not subject to ethical approval [14].

### 2.2 Study Population

PC patients treated with RT or CT from 2004 to 2016 were filtrated and extracted from the SEER database. PC diagnosis was based on the International Classification of Diseases 10th revision (ICD-10) criteria. The selection criteria were as follows: (1) clinicopathological evidence confirming PC with the prostate as the only primary site of the tumor; (2) complete clinical and pathological information available from 2004 to 2016; (3) follow-up duration of at least 1 month; (4) all patients had received RT or CT; (5) tumor stage is localized; (6) patients were male. The exclusion criteria were as follows: (1) patients with incomplete follow-up information; (2) participants with multiple primary tumors; (3) female patients; (4) unknown tumor grade, surgical status, race, marital status, Gleason score, and prostate-specific antigen (PSA).

### 2.3 Participant Variables

Participants’ variables included age at diagnosis (the optimal cut-off value of age was determined by X-tile 3.6.1 software (Yale University, New Haven, CT, USA), which divided the participants into 36–73 years old and ≥74 years old), year of diagnosis (2004–2009, 2010–2016), marital status (married, unmarried), race (white, black, others), surgery (yes, no evidence), tumor grade (Ⅰ, Ⅱ, Ⅲ, Ⅳ, with grade Ⅳ as undifferentiated, grade Ⅲ as poorly-differentiated, grade Ⅱ as moderately-differentiated, and grade Ⅰ defined as well-differentiated) [15], PSA (<10 ng/mL, 10–20 ng/mL, >20 ng/mL), Gleason score (<7, 7, >7). PSA is a serine protease enzyme produced primarily by the prostate gland. PSA levels in the blood are used as a biomarker for PC diagnosis and management [16, 17]. The Gleason score is a method for histological grading of PC. A Gleason score lower than 7 is mainly well-formed glands. A Gleason score equal to 7 is primarily poorly-formed, fused, cribriform glands, and well-formed glands have a lower composition. Gleason scores higher than 7, only poorly-formed, fused, cribriform glands, or lacks gland formation [18, 19].

CVD was the primary endpoint, measured from the time of PC diagnosis to the time of death from cardiovascular disease. Events other than CVD were considered competing events. The ICD-10 defines CVD as death from hypertension without heart disease (I10, I12), cardiopathy (I00–I09, I11, I13, I20–I51), atherosclerosis (I70), cerebrovascular disease (I60–I69), aortic aneurysm and dissection (I71) and arteriolar, capillary and other arterial diseases (I72–I78). Patients who did not survive the last follow-up or lost to follow-up before the end of the observation period were considered censored observations [20].

### 2.4 Construction of the Nomogram

The entire queue was stochastically divided into the training queue, and an internal validation queue in a ratio of 7:3. The differences in baseline data between the training and validation queues were analyzed and compared using the χ^2^ test. Univariate competitive risk analysis was conducted for preliminary screening in the training cohort. The multivariable competitive risk model included variables with significant differences from this screening. The multivariate competitive risk model was then used to select prognostic factors [21]. A nomogram was drawn based on the multivariable competitive risk analysis [22].

### 2.5 Nomogram Verification and Calibration

Concordance index (C-index) and calibration curve multiple validations were utilized to evaluate the accuracy of the nomogram [23]. The C-index was employed to estimate the consistency between the predicted result and the actual observation. The C-index value spans the interval from 0.5 to 1.0, where 0.5 indicates a random result, and 1.0 indicates a perfectly accurate prediction. The calibration curve was constructed by comparing the predicted and observed survival. The accuracy of the model increases as the expected curve approaches the actual curve.

### 2.6 Establishment of Risk Stratification

The risk stratification of PC patients was established using the final total score of each patient’s nomogram. New Haven, CT: Yale University, X-tile3.6.1 software was employed in the training cohort to identify the optimal cut-off points for the total score of each nomogram. The risk stratification was divided into a low-risk group (0–93 points), an intermediate-risk group (94–188 points), and a high-risk group (>193 points). The log-rank test and Kaplan-Meier (KM) survival analysis compared the statistical differences between the three risk groups in the training cohort. X-tile, a software developed by Yale University in 2004, is extensively used to identify the optimal cut-off point [24].

### 2.7 Statistical Method

The differences in baseline data across the training and validation cohorts were compared using the χ^2^ test in R software 3.5.1 (R Core Team, Vienna, Austria). The Fine-Gray competing risk analysis was conducted using R software 3.5.1. Based on the competitive risk analysis, a competitive risk prediction model was constructed, and the calibration curve of the nomogram model was described. C-index values were calculated for the training and internal verification cohorts. Sensitivity analysis was used to verify the robustness of the results. Statistical analyses, including the log-rank test and KM survival analysis (*p* < 0.05), were performed using SPSS 26.0 (IBM Corp., Chicago, IL, USA).

## 3. Results

### 3.1 Patient Characteristics

Data were extracted from the SEER database of patients diagnosed with PC and treated with RT or CT between 2004 and 2016. The study included 120,908 eligible men with PC who were followed for a median of 81 months (SD 0.2 months). Most patients were 36–73 years old (93,006, 76.9%), married (89,968, 74.4%), and white (91,052, 75.3%). The number of patients diagnosed in the two time periods of 2004–2009 and 2010–2016 was similar, and the number of patients diagnosed in 2004–2009 was slightly higher (61,881, 51.2%). Tumor grade Ⅱ (57,185, 47.3%) and grade Ⅲ (57,425, 47.5%) were the most common tumors, followed by grade I (6093, 5.04%). The majority of tumors were unilateral (120,373, 99.6%). The highest proportion of the Gleason score was <7 (49,474, 40.9%) or =7 (48,386, 40.0%), while 72.8% of patients had PSA <10 ng/mL, 17.7% exhibited PSA 10–20 ng/mL, and only 9.52% possessed PSA >20 ng/mL (Table 1).

**Table 1.** **Baseline data of 120,908 men with PC treated using RT or CT**.

Characteristics		Number (%)
Total		120,908
Age at diagnosis		
	36–73	93,006 (76.9%)
	≥74	27,902 (23.1%)
Marital status		
	Married	89,968 (74.4%)
	Unmarried	30,940 (25.6%)
Race		
	Other^a^	6781 (5.61%)
	White	91,052 (75.3%)
	Black	23,075 (19.1%)
Year of diagnosis		
	2004–2009	61,881 (51.2%)
	2010–2016	59,027 (48.8%)
Tumor grade		
	I	6093 (5.04%)
	II	57,185 (47.3%)
	III	57,425 (47.5%)
	IV	205 (0.17%)
Tumor laterality		
	Left side	139 (0.11%)
	Right side	216 (0.18%)
	Unilateral	120,373 (99.6%)
	Bilateral	180 (0.15%)
Surgery		
	Yes	4345 (3.59%)
	No	116,563 (96.4%)
PSA (ng/mL)		
	<10	87,980 (72.8%)
	10–20	21,414 (17.7%)
	<20	11,514 (9.52%)
Gleason score		
	<7	49,474 (40.9%)
	7	48,386 (40.0%)
	>7	23,048 (19.1%)

Abbreviations: PSA, prostate-specific antigen; PC, prostate cancer; RT, radiotherapy; CT, chemotherapy. ^a^ “Other” includes American Indians, Alaska Natives, Asians, and Pacific Islanders.

### 3.2 Variable Screening and Sensitivity Analysis

In the univariate competitive risks analysis, marital status, age at diagnosis, race, year of diagnosis, tumor grade, PSA, and Gleason score were all significantly associated with CVD in patients with PC after RT or CT (all *p* < 0.001). Neither surgery nor tumor laterality was significantly associated with CVD in these patients (both *p* > 0.05) (**Supplementary Table 1**).

Specifically, a PSA value of 10–20 ng/mL (crude hazard ratio (HR): 1.53, 95% confidence interval (CI): 1.43–1.64, *p* < 0.001), >20 ng/mL (crude HR: 1.60, 95% CI: 1.45–1.74, *p* < 0.001), and Gleason scores = 7 (crude HR: 1.36, 95% CI: 1.28–1.45, *p* < 0.001) and >7 (crude HR: 1.74, 95% CI: 1.62–1.88, *p* < 0.001) were associated with higher CVD risk in PC patients undergoing RT or CT (**Supplementary Table 2**).

Sensitivity analysis was adjusted for confounding variables to ascertain the influence of PSA and Gleason scores on RT or CT in patients with PC on CVD risk. In Model 1, robust adjusted HRs were observed for PSA scores of 10–20 ng/mL (crude HR: 1.28, 95% CI: 1.20–1.36, *p* < 0.001), >20 ng/mL (crude HR: 1.34, 95% CI: 1.24–1.45, *p* < 0.001), and Gleason scores = 7 (crude HR: 1.19, 95% CI: 1.13–1.26, *p* < 0.001) and >7 (crude HR: 1.36, 95% CI: 1.27–1.46, *p* < 0.001) (Table 2, **Supplementary Table 3**).

**Table 2.** **Sensitivity analysis of the effects of PSA and Gleason scores on CVD risk**.

Variables		Crude HR		Model 1^a^		Model 2^b^	
		HR (95% CI)	*p*-value	HR (95% CI)	*p*-value	HR (95% CI)	*p*-value
PSA (ng/mL)							
	<10	Reference		Reference		Reference	
	10–20	1.53 (1.44–1.62)	<0.001	1.28 (1.20–1.36)	<0.001	1.27 (1.21–1.35)	<0.001
	>20	1.63 (1.51–1.75)	<0.001	1.34 (1.24–1.45)	<0.001	1.35 (1.25–1.46)	<0.001
Gleason score							
	<7	Reference		Reference		Reference	
	7	1.37 (1.29–1.44)	<0.001	1.19 (1.13–1.26)	<0.001	1.09 (0.96–1.23)	0.210
	>7	1.80 (1.69–1.92)	<0.001	1.36 (1.27–1.46)	<0.001	1.23 (1.07–1.41)	0.004

Abbreviations: CVD, cardiovascular death; PSA, prostate-specific antigen; CI, confidence interval; HR, hazard ratio. ^a^ HRs were adjusted for factors of social demographic characteristics, including age at diagnosis, marital status, year of diagnosis, race, PSA, and Gleason score. ^b^ HRs were adjusted across all variables in the baseline, including age at diagnosis, marital status, race, year of diagnosis, PSA, Gleason score, tumor grade, tumor laterality, and surgery.

After adjusting for all variables in Model 2, the adjusted HR for the PSA and Gleason scores remained stable (PSA 10–20 ng/mL adjusted HR: 1.27, 95% CI: 1.21–1.35, *p* < 0.001; PSA >20 ng/mL adjusted HR: 1.35, 95% CI: 1.25–1.46, *p* < 0.001; Gleason score = 7 adjusted HR: 1.09, 95% CI: 0.96–1.23, *p* = 0.210; Gleason score >7 adjusted HR: 1.23, 95% CI: 1.07–1.41, *p* = 0.004) (Table 2, **Supplementary Table 4**).

### 3.3 Development and Verification Process of Each Nonogram

The training queue comprised 84,636 patients, while the validation cohort comprised 36,272 patients. No significant differences in baseline characteristics were observed between the training and validation queues (*p* > 0.05) (**Supplementary Table 5**).

**Fig. 1.**
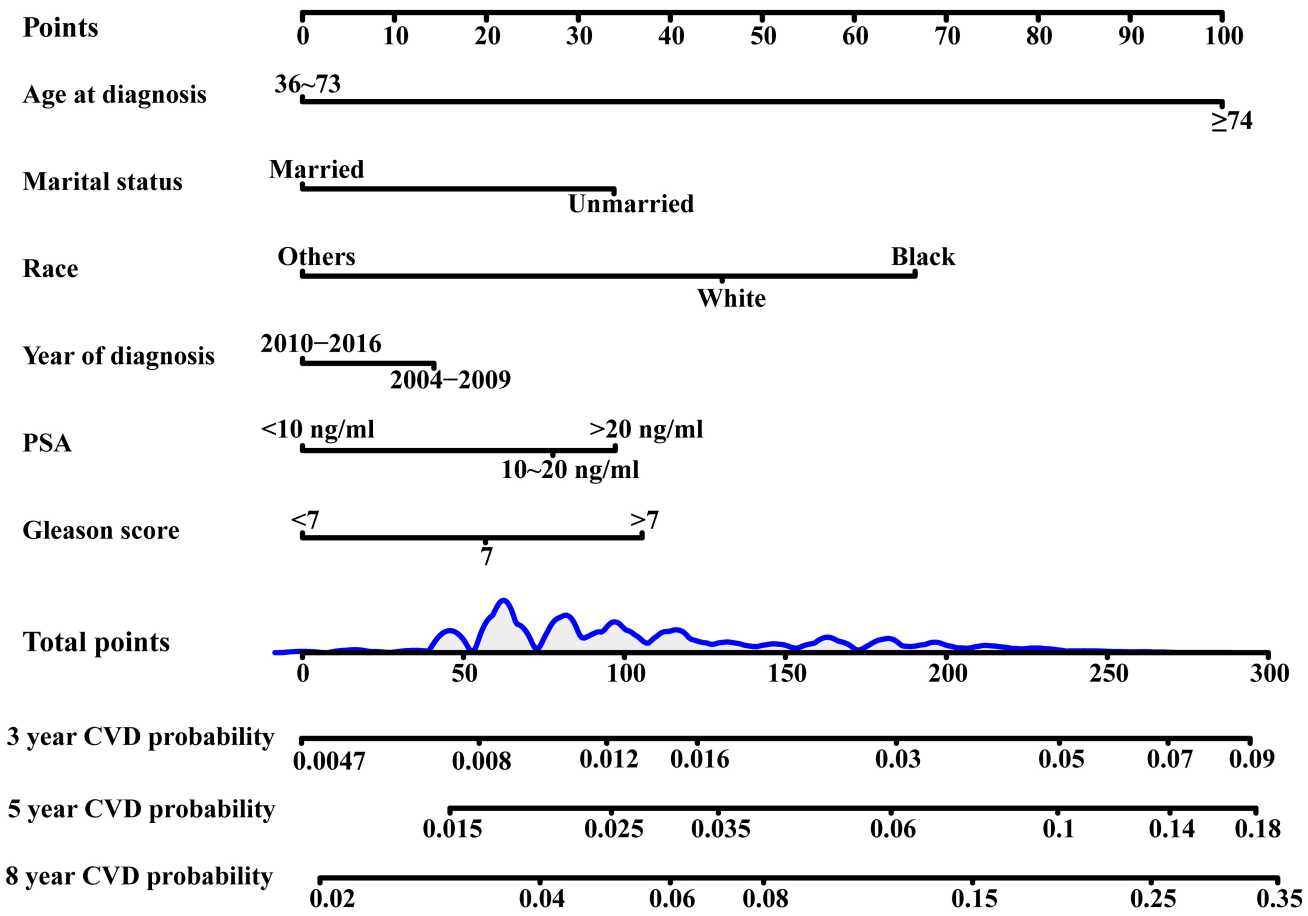
**Nomogram of 3-year, 5-year and 8-year CVD predictions**. Abbreviations: CVD, cardiovascular death; PSA, prostate-specific antigen.

In both univariate and multivariate competitive risk analyses of the training queue, age at diagnosis, marital status, race, year of diagnosis, PSA value, and Gleason score were associated with the risk of CVD (**Supplementary Table 2**). Based on these analyses, we generated a nomogram to predict CVD risk at 3, 5, and 8 years in PC patients treated with RT or CT. Age was given a maximum rating of 100 points, followed by race, Gleason score, marital status, PSA value, and year of diagnosis (**Supplementary Table 6**). This nomogram calculated the 3-year, 5-year, and 8-year risk of CVD in PC patients following treatment with RT or CT. The 3-year, 5-year, and 8-year CVD risk was calculated by summing the scores of the six variables (Fig. 1).

**Fig. 2.**
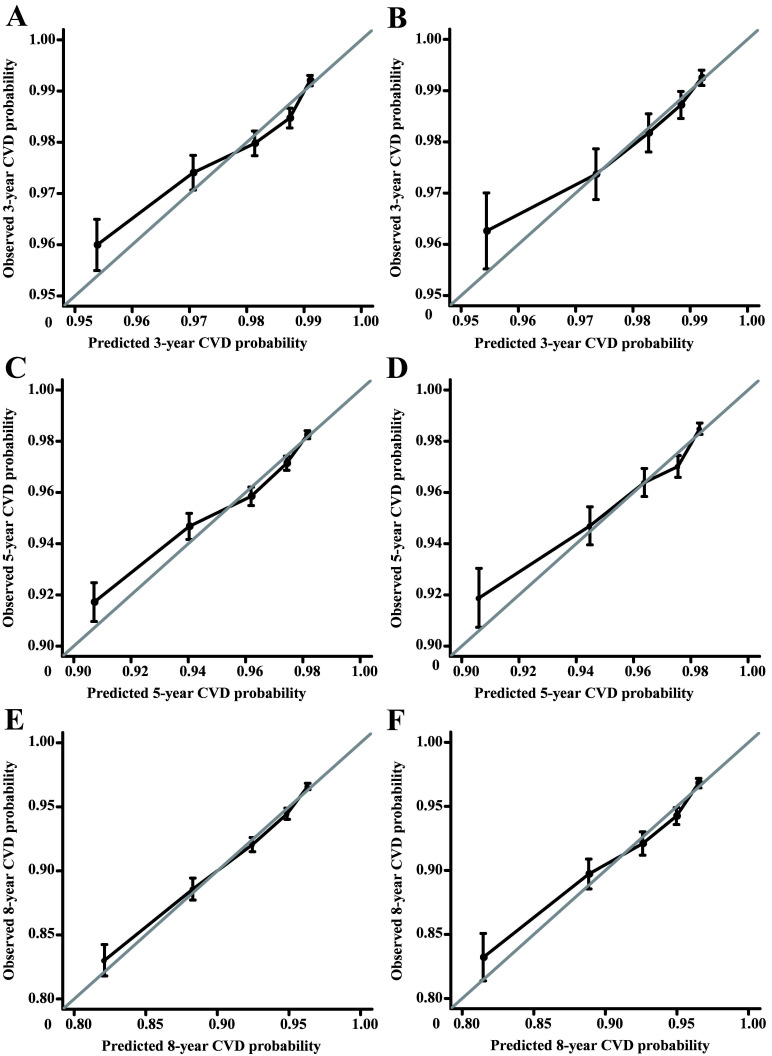
**Calibration curves of the training cohort and internal validation cohort**. (A) The training cohort was validated over 3 years. (B) The validation cohort was validated over 3 years. (C) The training cohort was validated over 5 years. (D) The validation cohort was validated over 5 years. (E) The training cohort was validated over 8 years. (F) The validation cohort was validated over 8 years. The 45° gray line signifies perfect alignment between the actual (Y-axis) and predicted survival outcomes (X-axis). Closer adherence to this line indicates higher model accuracy. Abbreviations: CVD, cardiovascular death.

The C-index of the model training cohort was 0.66 (95% CI: 0.66–0.67); the C-index of the validation cohort was 0.67 (95% CI: 0.65–0.68). The calibration curve consequences of the training and validation queues showed that the incidence of CVD at 3, 5, and 8 years was drawn near the actual CVD risk, suggesting that the nomogram model had good predictive capacity (Fig. 2).

### 3.4 Risk Stratification

**Fig. 3.**
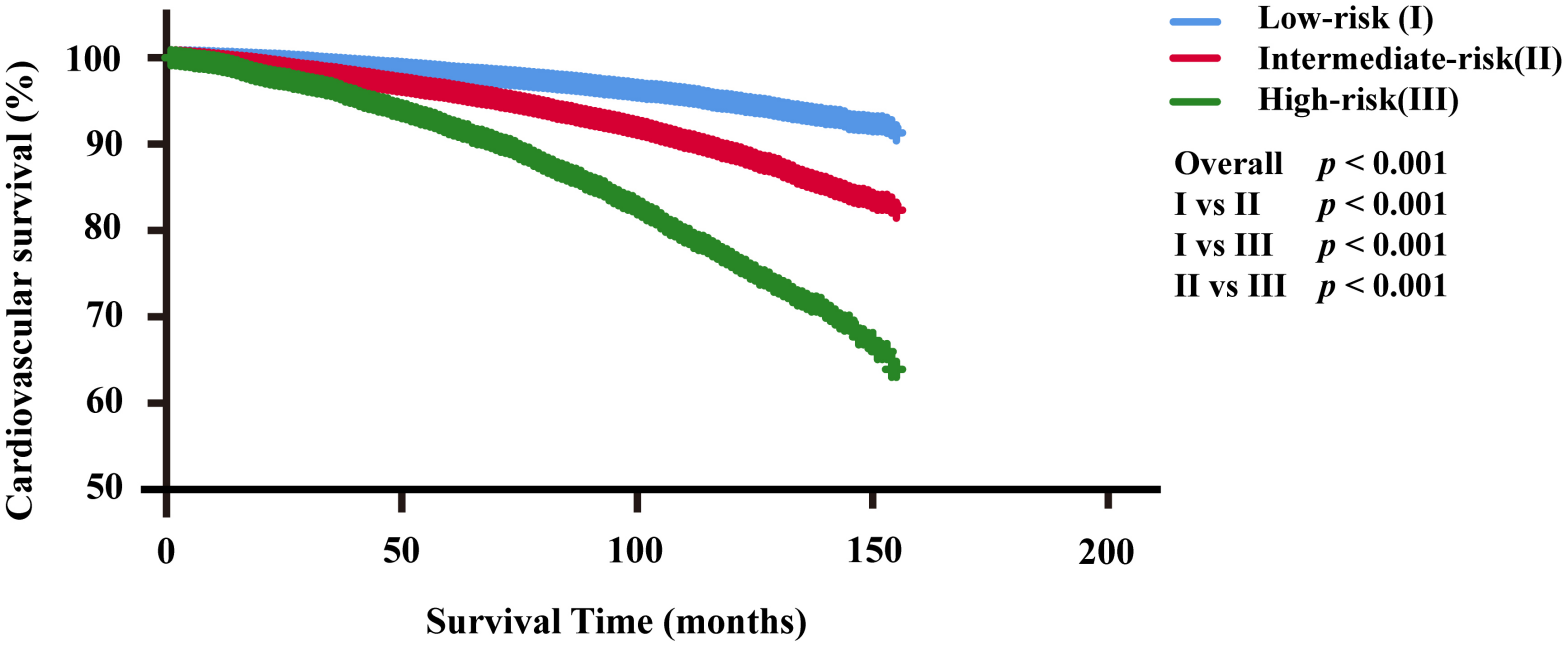
**Cardiovascular survival analysis for the three risk groups**. Abbreviations: I, low-risk group; II, intermediate-risk group; III, high-risk group.

The CVD risk stratification of PC patients receiving RT or CT was determined based on the total score predicted by the nomogram, categorizing patients as low-risk, intermediate-risk, and high-risk groups. The low-risk group was classified with 0–93 points, the intermediate-risk group with 94–188 points, and the high-risk group with >193 points. Fig. 3 shows that the CVD risk in the low-risk group was significantly lower in the training cohort than in the other two groups, with the high-risk group exhibiting the highest CVD risk (overall *p*-value and pairwise comparison *p*-value both <0.001), suggesting that this risk stratification effectively reflects the CVD risk for PC patients who received RT or CT (Fig. 3).

## 4. Discussion

In this population-based research, we evaluated the effect of PSA and Gleason scores on CVD risk in PC patients who had received RT or CT for the first time. Our results demonstrated that the PSA and Gleason scores were associated with CVD in PC patients who had received RT or CT.

Previous studies consistently indicate that RT or CT can lead to cardiovascular toxicity in PC patients [2, 5]. Consequently, our study focused on identifying risk factors for CVD, specifically in PC patients who had undergone RT or CT. It is important to clarify that our analysis did not consider RT or CT an exposure factor.

PSA plays a vital role in the early screening and diagnosis of PC. High concentrations of PSA are generally associated with the presence of or high risk of PC [25, 26, 27]. Elevated PSA levels are generally associated with the presence ofare usually associated with PC and a higher risk of the disease [28, 29]. Meanwhile, previous studies have suggested a possible association between PSA and the cardiovascular system [28]. Several studies have reported elevated PSA levels during acute myocardial infarction [29, 30]. There are also studies showing a significant correlation between higher rates of PSA and the occurrence of non-ST-elevation myocardial infarction [31]. Our findings indicate that PC patients who received RT or CT and had PSA levels ≥10 ng/mL faced a higher risk of CVD compared to those with PSA levels <10 ng/mL. Previous investigations have primarily focused on the relationship between PSA and overall survival (OS) and cancer-specific survival (CSS) in PC while overlooking its effect on CVD [32, 33, 34, 35, 36]. Consequently, PC patients with PSA levels ≥10 ng/mL require enhanced cardiovascular monitoring and management. Clinical research has shown that reductions in PSA levels are associated with decreased incidence of cardiovascular adverse events, such as ischemic heart disease, which aligns with our findings [37].

The Gleason score is a critical prognostic indicator for patients with PC [34, 38]. An increase in Gleason score is directly associated with several histopathological and clinical endpoints, including lymphovascular invasion, tumor volume, positive resection margin, pathology stage, and the risk of prostate abduction and metastasis [39, 40]. Additionally, the Gleason score is commonly used in constructing prognostic nomograms for PC and is generally considered one of the independent factors related to PC prognosis [41, 42, 43]. Nevertheless, most previous investigations have focused on the relationship between the Gleason score and OS and CSS in PC, often overlooking its impact on CVD [42]. Our study revealed that PC patients who had received RT or CT and had a Gleason score greater than 7 faced a higher risk of CVD. These findings suggest that PC patients with high Gleason scores who received RT or CT require vigilant monitoring and management of cardiovascular adverse events.

Most previous prediction models for CVD risk in PC patients have predominantly included those treated with androgen therapy, with limited focus on patients treated using RT or CT [44, 45, 46]. Further, the effects of PSA and Gleason scores are often ignored when evaluating CVD risk factors for PC patients. Thus, a comprehensive consideration of these and other risk factors can enhance the robustness and personalization of CVD prediction models. Our results address the limitations of existing models by incorporating the PSA and Gleason scores into a visual nomogram that depicts their impact on CVD risk. Although our predictive nomogram has areas for improvement, integrating the PSA and Gleason scores with other clinical variables can aid in personalized CVD risk assessment and guide clinical prevention strategies.

Higher PSA levels and Gleason scores typically reflect advanced disease progression, severity, and poorer prognosis, leading to more aggressive treatments and increased cardiovascular burden, which elevates CVD risk [2, 5, 6, 27, 47]. Our study identified an association between high PSA levels, elevated Gleason scores, and an increased risk of CVD in PC patients. This suggests that the PSA levels and Gleason scores can be critical indicators for assessing CVD risk in PC patients. Hence, when evaluating the cardiovascular health of a patient, physicians should consider these factors and implement appropriate preventive measures and management strategies to mitigate CVD risk.

A significant advantage of our research is the large sample size. Our research is one of the largest and earliest investigations to evaluate the impact of PSA levels and Gleason scores on CVD risk in PC patients who had received RT or CT treatment. However, several limitations should be acknowledged. First, although we used a training set to build the model and validated it using a separate validation set, all data were sourced from the SEER database, which may introduce inherent biases. Second, cardiovascular events are typically the result of a multifactorial process. The SEER database lacks information on cardiovascular comorbidities, common risk factors, and specific treatment regimens, including systemic therapy and androgen deprivation therapy. Subsequently, this limits our ability to further analyze, evaluate, and generalize our findings. Third, given the extensive duration of this retrospective study, there are inevitable confounding factors. For instance, advancements in treating and managing PC and CVD during the follow-up period may confound the results. Thus, future research should involve large cohort studies to validate our findings.

## 5. Conclusions

PSA levels ≥10 ng/mL and Gleason scores >7 may be associated with an increased risk of CVD in PC patients after RT or CT. In addition, we have successfully developed a nomogram to visually represent the effect of PSA levels and Gleason scores on CVD risk. Consequently, preventive strategies and clinical interventions should be actively adopted for patients meeting the above conditions to alleviate the adverse effects of cardiovascular diseases and reduce the risk of CVD. Our research conclusion still needs to be verified by the next prospective study.

## Data Availability

The datasets are publicly available from the SEER database (http://seer.cancer.gov).

## Abbreviations

CVD, cardiovascular death; PC, prostate cancer; RT, radiotherapy; CT, chemotherapy; PSA, prostate-specific antigen; C-index, concordance indexes; SEER, the Surveillance, Epidemiology, and End Results; CI, confidence interval; HR, hazard ratio; OS, overall survival; CSS, cancer-specific survival.

## Supplementary Material

Supplementary material associated with this article can be found, in the online version, at https://doi.org/10.31083/RCM24940.
