# Supplementary material for: The Impact of Prostate-Specific Antigen and Gleason Scores on Cardiovascular Death in Prostate Cancer Patients after Radiotherapy or Chemotherapy: A Population-Based Study
Source: Rev Cardiovasc Med. 2025 Feb 19;26(2):24940. doi: 10.31083/RCM24940 (PMC11868894; doi:10.31083/RCM24940)
Supplement: Supplementary file 1 [file 2153-8174-26-2-24940-s1.docx]

Supplementary Material

**Supplementary Table 1.** The univariate competitive risks analysis of 120,908 men with PC treated with RT or CT(the whole cohort).

**Supplementary Table 2.** The univariate and multivariate competitive risk analysis of training queue.

**Supplementary Table 3.** Model 1 of sensitivity analysis.

**Supplementary Table 4.** Model 2 of sensitivity analysis.

**Supplementary Table 5.** The baseline characteristics of the training queue and the validation queue.

**Supplementary Table 6.** The specific score of each variable in the nomogram.

**Supplementary Table 1.** The univariate competitive risks analysis of 120,908 men with PC treated with RT or CT (the whole cohort).

| **Variables** | **HR （95%CI）** | ***p-value*** |
| --- | --- | --- |
| **Age at diagnosis** |  |  |
| 36-73 | Reference |  |
| ≥74 | 2.657 (2.531-2.789) | <0.001 |
| **Marital status** |  |  |
| Married | Reference |  |
| Unmarried | 1.437 (1.364-1.514) | <0.001 |
| **Race** |  |  |
| Other ^a^ | Reference |  |
| White | 1.316 (1.167-1.483) | <0.001 |
| Black | 1.468 (1.291-1.669) | <0.001 |
| **Year of diagnosis** |  |  |
| 2010-2016 | Reference |  |
| 2004-2009 | 1.212 (1.134-1.295) | <0.001 |
| **Tumor grade** |  |  |
| Ⅰ | Reference |  |
| Ⅱ | 1.082 (0.837-1.399) | 0.550 |
| Ⅲ | 1.617 (1.252-2.088) | <0.001 |
| Ⅳ | 2.032 (1.224-3.372) | 0.006 |
| **Surgery** |  |  |
| Yes | Reference |  |
| No | 1.013 (0.890-1.154) | 0.840 |
| **PSA (ng/mL)** |  |  |
| <10 | Reference |  |
| 10-20 | 1.527 (1.438-1.620) | <0.001 |
| >20 | 1.628 (1.511-1.753) | <0.001 |
| **Gleason score** |  |  |
| <7 | Reference |  |
| 7 | 1.365 (1.292-1.443) | <0.001 |
| >7 | 1.801 (1.690-1.918) | <0.001 |
| **Tumor laterality** |  |  |
| Left side | Reference |  |
| Right side | 1.470 (0.288-7.514) | 0.640 |
| unilateral | 1.315 (0.331-5.223) | 0.700 |
| Bilateral | 1.909 (0.354-10.284) | 0.450 |

Abbreviations: PSA, prostate-specific antigen.

^a^ The other included American Indian, Alaska Native, Asian, Pacific Islander.

**Supplementary Table 2.** The univariate and multivariate competitive risk analysis of training queue.

| **Variables** | **Univariate analysis** | | **Multivariate analysis** | |
| --- | --- | --- | --- | --- |
|  | **HR （95%CI）** | ***p*** | **HR （95%CI）** | ***p-value*** |
| **Age at diagnosis** |  |  |  |  |
| 36-73 | Reference |  | Reference |  |
| ≥74 | 2.651 (2.503-2.808) | <0.001 | 2.586 (2.435-2.746) | <0.001 |
| **Marital status** |  |  |  |  |
| Married | Reference |  | Reference |  |
| Unmarried | 1.399 (1.315-1.489) | <0.001 | 1.373 (1.288-1.463) | <0.001 |
| **Race** |  |  |  |  |
| Other ^a^ | Reference |  | Reference |  |
| White | 1.374 (1.187-1.591) | <0.001 | 1.543 (1.333-1.786) | <0.001 |
| Black | 1.568 (1.341-1.833) | <0.001 | 1.910 (1.631-2.236) | <0.001 |
| **Year of diagnosis** |  |  |  |  |
| 2010-2016 | Reference |  | Reference |  |
| 2004-2009 | 1.210 (1.118-1.309) | <0.001 | 1.269 (1.172-1.374) | <0.001 |
| **Tumor grade** |  |  |  |  |
| Ⅰ | Reference |  | Reference |  |
| Ⅱ | 1.134 (0.834-1.541) | 0.420 | 1.025 (0.752-1.396) | 0.880 |
| Ⅲ | 1.669 (1.229-2.266) | 0.001 | 1.125 (0.798-1.586) | 0.500 |
| Ⅳ | 2.145 (1.189-3.872) | 0.011 | 1.282 (0.692-2.374) | 0.430 |
| **Tumor laterality** |  |  |  |  |
| Left side | Reference |  |  |  |
| Right side | 0.868 (0.161-4.694) | 0.870 |  |  |
| unilateral | 0.811 (0.306-0.151) | 0.670 |  |  |
| Bilateral | 1.270 (0.287-5.620) | 0.750 |  |  |
| **Surgery** |  |  |  |  |
| Yes | Reference |  |  |  |
| No | 1.012 (0.867-1.181) | 0.880 |  |  |
| **PSA (ng/mL)** |  |  |  |  |
| <10 | Reference |  | Reference |  |
| 10-20 | 1.529 (1.425-1.640) | <0.001 | 1.283 (1.193-1.379) | <0.001 |
| >20 | 1.589 (1.453-1.736) | <0.001 | 1.338 (1.220-1.467) | <0.001 |
| **Gleason score** |  |  |  |  |
| <7 | Reference |  | Reference |  |
| 7 | 1.362 (1.275-1.454) | <0.001 | 1.093 (0.942-1.268) | 0.240 |
| >7 | 1.744 (1.617-1.880) | <0.001 | 1.202 (1.019-1.417) | 0.029 |

Abbreviations: PSA, prostate-specific antigen.

^a^ The other included American Indian, Alaska Native, Asian, Pacific Islander.

**Supplementary Table 3.** Model 1 of sensitivity analysis.

| **Variables** | **HR （95%CI）** | ***p-value*** |
| --- | --- | --- |
| **Age at diagnosis** |  |  |
| 36-73 | Reference |  |
| ≥74 | 2.57 (2.44-2.70) | <0.001 |
| **Marital status** |  |  |
| Married | Reference |  |
| Unmarried | 1.42 (1.34-1.49) | <0.001 |
| **Race** |  |  |
| Other ^a^ | Reference |  |
| White | 1.48 (1.31-1.67) | <0.001 |
| Black | 1.78 (1.56-2.03) | <0.001 |
| **Year of diagnosis** |  |  |
| 2010-2016 | Reference |  |
| 2004-2009 | 1.28 (1.20-1.36) | <0.001 |
| **PSA (ng/mL)** |  |  |
| <10 | Reference |  |
| 10-20 | 1.28 (1.20-1.36) | <0.001 |
| >20 | 1.34 (1.24-1.45) | <0.001 |
| **Gleason score** |  |  |
| <7 | Reference |  |
| 7 | 1.19 (1.13-1.26) | <0.001 |
| >7 | 1.36 (1.27-1.46) | <0.001 |

Abbreviations: PSA, prostate-specific antigen.

^a^ The other included American Indian, Alaska Native, Asian, Pacific Islander.

**Supplementary Table 4.** Model 2 of sensitivity analysis.

| **Variables** | **HR （95%CI）** | ***p-value*** |
| --- | --- | --- |
| **Age at diagnosis** |  |  |
| 36-73 | Reference |  |
| ≥74 | 2.57 (2.44-2.70) | <0.001 |
| **Marital status** |  |  |
| Married | Reference |  |
| Unmarried | 1.42 (1.34-1.49) | <0.001 |
| **Race** |  |  |
| Other ^a^ | Reference |  |
| White | 1.48 (1.31-1.67) | <0.001 |
| Black | 1.78 (1.56-2.03) | <0.001 |
| **Year of diagnosis** |  |  |
| 2010-2016 | Reference |  |
| 2004-2009 | 1.27 (1.19-1.36) | <0.001 |
| **Tumor grade** |  |  |
| Ⅰ | Reference |  |
| Ⅱ | 0.98 (0.76-1.27) | 0.880 |
| Ⅲ | 1.091 (0.82-1.45) | 0.550 |
| Ⅳ | 1.192 (0.70-2.02) | 0.520 |
| **Surgery** |  |  |
| Yes | Reference |  |
| No | 1.02 (0.90-1.16) | 0.730 |
| **PSA (ng/mL)** |  |  |
| <10 | Reference |  |
| 10-20 | 1.27 (1.21-1.35) | <0.001 |
| >20 | 1.35 (1.25-1.46) | <0.001 |
| **Gleason score** |  |  |
| <7 | Reference |  |
| 7 | 1.09 (0.96-1.23) | 0.210 |
| >7 | 1.23(1.07-1.41) | 0.004 |
| **Tumor laterality** |  |  |
| Left side | Reference |  |
| Right side | 1.56 (0.31-7.82) | 0.590 |
| unilateral | 1.19 (0.30-4.66) | 0.810 |
| Bilateral | 1.85 (0.34-1.00) | 0.480 |

Abbreviations: PSA, prostate-specific antigen.

^a^ The other included American Indian, Alaska Native, Asian, Pacific Islander.

**Supplementary Table 5.** The baseline characteristics of the training queue and the validation queue.

| **Characteristics** | **Training queue** | **Validation queue** | ***p-value*** |
| --- | --- | --- | --- |
|  | **N=84636** | **N=36272** |  |
| **Age at diagnosis** |  |  | 0.396 |
| 36-73 | 65,047 (76.9%) | 27,959 (77.1%) |  |
| ≥74 | 19,589 (23.1%) | 8313 (22.9%) |  |
| **Marital status** |  |  | 0.132 |
| Married | 63,083 (74.5%) | 26,885 (74.1%) |  |
| Unmarried | 21,553 (25.5%) | 9387 (25.9%) |  |
| **Race** |  |  | 0.553 |
| White | 63,718 (75.3%) | 27,334 (75.4%) |  |
| Black | 16,202 (19.1%) | 6873 (18.9%) |  |
| Other ^a^ | 4716 (5.57%) | 2065 (5.69%) |  |
| **Year of diagnosis** |  |  | 0.157 |
| 2004-2009 | 43,430 (51.3%) | 18,451 (50.9%) |  |
| 2010-2016 | 41,206 (48.7%) | 17,821 (49.1%) |  |
| **Tumor grade** |  |  | 0.985 |
| Ⅰ | 4264 (5.04%) | 1829 (5.04%) |  |
| Ⅱ | 40,034 (47.3%) | 17,151 (47.3%) |  |
| Ⅲ | 40,192 (47.5%) | 17,233 (47.5%) |  |
| Ⅳ | 146 (0.17%) | 59 (0.16%) |  |
| **Tumor laterality** |  |  | 0.506 |
| Left side | 98 (0.12%) | 41 (0.11%) |  |
| Right side | 154 (0.18%) | 62 (0.17%) |  |
| unilateral | 84,267 (99.6%) | 36,106 (99.5%) |  |
| Bilateral | 117 (0.14%) | 63 (0.17%) |  |
| **Surgery** |  |  | 0.098 |
| Yes | 2992 (3.54%) | 1353 (3.73%) |  |
| No | 81,644 (96.5%) | 34,919 (96.3%) |  |
| **PSA (ng/mL)** |  |  | 0.940 |
| <10 | 61,586 (72.8%) | 26,394 (72.8%) |  |
| 10-20 | 15,004 (17.7%) | 6410 (17.7%) |  |
| >20 | 8046 (9.51%) | 3468 (9.56%) |  |
| **Gleason score** |  |  | 0.210 |
| <7 | 34,521 (40.8%) | 14,953 (41.2%) |  |
| 7 | 33,885 (40.0%) | 14,501 (40.0%) |  |
| >7 | 16,230 (19.2%) | 6818 (18.8%) |  |

Abbreviations: PSA, prostate-specific antigen.

^a^ The other included American Indian, Alaska Native, Asian, Pacific Islander.

**Supplementary Table 6.** The specific score of each variable in the nomogram.

| **Variables** | **scores** |
| --- | --- |
| **Age at diagnosis** |  |
| 36-73 | 0 |
| ≥74 | 100 |
| **Marital status** |  |
| Married | 0 |
| Unmarried | 34 |
| **Race** |  |
| Other ^a^ | 0 |
| White | 46 |
| Black | 67 |
| **Year of diagnosis** |  |
| 2010-2016 | 0 |
| 2004-2009 | 14 |
| **PSA (ng/mL)** |  |
| <10 | 0 |
| 10-20 | 27 |
| >20 | 34 |
| **Gleason score** |  |
| <7 | 0 |
| 7 | 20 |
| >7 | 37 |

Abbreviations: PSA, prostate-specific antigen.

^a^ The other included American Indian, Alaska Native, Asian, Pacific Islander.
